# CircDUSP16 promotes the tumorigenesis and invasion of gastric cancer by sponging miR-145-5p

**DOI:** 10.1007/s10120-019-01018-7

**Published:** 2019-11-27

**Authors:** Zizhen Zhang, Chaojie Wang, Yeqian Zhang, Site Yu, Gang Zhao, Jia Xu

**Affiliations:** Department of Gastrointestinal Surgery, Ren Ji Hospital, School of Medicine, Shanghai Jiao Tong University, No. 160 Pu Jian Road, Shanghai, 200127 China

**Keywords:** Gastric cancer, circDUSP16, miR-145-5p, Growth, Invasion

## Abstract

**Background:**

Circular RNAs (circRNAs) as a novel subgroup of non-coding RNAs act a critical role in the pathogenesis of gastric cancer (GC). However, the underlying mechanisms by which hsa_circ_0003855 (circDUSP16) contributes to GC are still undocumented.

**Materials:**

The differentially expressed circRNAs were identified by GEO database. The association of circDUSP16 or miR-145-5p expression with clinicopathological features and prognosis in GC patients was analyzed by FISH and TCGA-seq data set. Loss- and gain-of-function experiments as well as a xenograft tumor model were performed to assess the role of circDUSP16 in GC cells. circDUSP16-specific binding with miR-145-5p was confirmed by bioinformatic analysis, luciferase reporter, and RNA immunoprecipitation assays.

**Results:**

The expression levels of circDUSP16 were markedly increased in GC tissue samples and acted as an independent prognostic factor of poor survival in patients with GC. Knockdown of circDUSP16 repressed the cell viability, colony formation, and invasive potential in vitro and in vivo, but ectopic expression of circDUSP16 reversed these effects. Moreover, circDUSP16 possessed a co-localization with miR-145-5p in the cytoplasm, and acted as a sponge of miR-145-5p, which attenuated circDUSP16-induced tumor-promoting effects and IVNS1ABP expression in GC cells. MiR-145-5p had a negative correlation with circDUSP16 expression and its low expression was associated with poor survival in GC patients.

**Conclusions:**

CircDUSP16 facilitates the tumorigenesis and invasion of GC cells by sponging miR-145-5p, and may provide a novel therapeutic target for GC.

**Electronic supplementary material:**

The online version of this article (10.1007/s10120-019-01018-7) contains supplementary material, which is available to authorized users.

## Introduction

Gastric cancer (GC) is one of the most common malignancies worldwide. Despite the decreased incidence of GC, it is still the leading cause of cancer-related mortality [1]. Every effort has been made to improve the pathogenesis and therapeutic strategies of GC [2], such as eradication of *Helicobacter pylori* [3], but GC is generally diagnosed at an advanced stage and its prognosis is poor due to tumor invasiveness [4]. Substantial evidence shows that deregulated expression of non-coding RNAs (ncRNAs) is linked to the progression of GC [5, 6]. Thus, it is indispensable to identify novel biomarkers for early detection of GC.

Circular RNAa (circRNAs), a new subtype of ncRNAs, have covalently closed loop structures with a back splice site between 5′- and 3′-end and exhibit higher conservativity than the corresponding linear RNAs duo to resistance to RNase R [7]. Mounting data indicated that circRNAs act critical roles in multiple molecular mechanisms including tumor biomarkers, regulating gene expression, and sponging miRNAs in cancer [8-10]. Circ-DONSON [8], circAGO2 [9], circAKT3 [11], circNRIP1 [12], and circDLST [13] are upregulated in GC tissues samples, and their increased expression is associated with TNM stage and poor prognosis in patients with GC [8, 11, 13]. CircAKT3 and circDLST act as the sponges of miR-198/-502-5p to favor the tumorigenesis and cisplatin resistance in GC cells [11, 13]. In addition, circ-KIAA1244 [14], circPSMC3 [15], and circFAT1(e2) [16] are downregulated in GC tissues and plasmas, and their decreased expression is related to tumor invasiveness and poor survival in GC patients [14-16]. These circRNAs may provide potential biomarkers for the treatment of GC.

MicroRNAs (miRNAs) as another subgroup of small ncRNAs negatively regulate their target genes and act as oncogenes or tumor suppressors in GC [17, 18]. Previous studies showed that decreased expression of miR-145-5p caused by promoter methylation is a prognostic factor for endometrial cancer, and it suppresses the growth of laryngeal carcinoma by targeting FSCN1 [19, 20]. Exosomes delivered miR-145-5p also represses the progression of pancreatic adenocarcinoma and ovarian cancer [21, 22]. Moreover, miR-145-5p act as a tumor suppressor in GC by targeting N-cadherin and ZEB2 [23]. These studies indicate that miR-145-5p may be a potential target in cancer.

In the present study, we identified a new hsa_circ_0003855 (circDUSP16) and found that its upregulation was associated with poor survival in patients with GC. Ectopic expression of circDUSP16 promoted cell viability, colony formation, and tumor invasion in vitro and in vivo by sponging miR-145-5p. MiR-145-5p, co-localized with circDUSP16 in the cytoplasm, had a negative correlation with circDUSP16 expression, and counteracted circDUSP16-induced GC-promoting effects. Our findings might provide a prognostic biomarker for GC patients.

## Materials and methods

### Clinical samples

A tissue microarray (No. ST810b) including 40 paired GC tissue samples was purchased from Alenabio Biotechnology Co., Ltd (Xi’an, China). The clinicopathological and prognostic data for GC patients as well as miR-145-5p and IVNS1ASBP expression levels were downloaded from TCGA RNA-seq data set (https://xena.ucsc.edu/). The patients did not receive any chemotherapy, and the protocols were approved by the Ethics Committee of Renji Hospital of Shanghai Jiao Tong University.

### Bioinformatic analysis

The differentially expressed circRNAs were identified between GC and adjacent normal tissues using GSE78092 data (https://www.gcbi.com.cn/gclib/html/index); CircDUSP16-specific binding with miRNAs was identified using Circular RNA Interactome (https://circinteractome.nia.nih.gov/index.html) according to the binding stringency; the target genes of miR-145-5p were identified using the TargetScanHuman7.1 (https://www.targetscan.org/vert_71/) according to the cumulative weighted context scores.

### RNA fluorescence in situ hybridization (FISH) analysis

Digoxin-labeled probe sequences for hsa_circ_0003855 (circDUSP16) (5′- ACTGGACTGAAAGCCTGCAAGCAGGTGA-3′) and Biotin-labeled probe sequences for miR-145-5p (5′-AGGGATTCCTGGGAAAACTGGAC-3′) were used to analyze the expression levels and cellular localization of circDUSP16 in GC cells and tissue samples. The detailed description of FISH analysis was conducted as previously reported [6].

### Cell culture

GC cell lines used in this study were cultured in DMEM medium supplemented with 10% heat-inactivated FBS in a humidified atmosphere containing 5% CO2 at 37 °C.

### Quantitative real-time PCR (qRT-PCR) analysis

RNA was isolated from the GC cells using Trizol reagent (Invitrogen) according to the manufacturer’s instructions. To assess the expression levels of circDUSP16, DUSP16, miR-145-5p, and IVNS1ABP in GC tissue samples and cell lines, we performed a qRT–PCR analysis. GAPDH or U6 was used as an endogenous control. The designed primer sequences of circDUSP16, DUSP16, miR-145-5p, and IVNS1ABP were listed in the supplementary Table S1. The detailed description of qRT-PCR analysis was conducted as previously reported [4].

### Western blot analysis

GC cell lines were harvested and extracted using lysis buffer. The primary antibodies against PCNA (ab15497, Abcam, Cambridge, MA, USA), MMP-2 (ab92536, Abcam, Cambridge, MA, USA), and IVNS1ABP (GTX118817, GeneTex, Shanghai, China) were diluted at a ratio of 1:1000 according to the instructions and incubated overnight at 4 ℃. The specific details for Western blot analysis were conducted as previously described [4].

### Actinomycin D and RNase R treatment

Actinomycin D or RNase R treatment was performed as previously described [6].

### Luciferase reporter assay

GC cell lines were seeded into 24-well plates. After 24 h incubation, 6 ng of pmirGLO report vector containing wild type (WT) or mutant (Mut) circDUSP16 and IVNS1ABP 3′UTR was co-transfected with miR-145-5p mimic into BGC-823 and SGC-7901 cell lines. After the transfection for 48 h, luciferase activities were examined with a dual-luciferase Reporter System (Promega).

### Plasmid, shRNA, and miR-145-5p mimic

Lentivirus-mediated sh-circDUSP16 (target sequence: ACCTGCTTGCAGGCTTT CA GT), plasmid-mediated circDUSP16, miR-145-5p mimic, and miR-NC were purchased from Genepharma (Shanghai, PR, China). GC cells were planted in 6-well plates 24 h prior to sh-circDUSP16, circDUSP16, and miR-145-5p mimic transfection with 50-70% confluence in GC cells according to the manufacture instructions.

### MTT, colony formation, and transwell assays

MTT, colony formation, and transwell assays were conducted as previously reported [4].

### RNA immunoprecipitation (RIP)

RIP assay was conducted as previously reported [6].

### Animal experiments

Six-week-old female immune-deficient nude mice (BALB/c-nu) were injected subcutaneously with 1 × 10^7^ MKN-45 cells stably transfected with sh-circDUSP16 or sh-NC. Mice were monitored daily and developed a subcutaneous xenograft tumor. The tumor volumes were detected with a caliper using the formula: volume = (length × width^2^)/2. The animal study was approved by the Ethics Committee of Renji Hospital.

### Statistical analysis

Statistical analyses were carried out by SPSS 20.0 (IBM, SPSS, Chicago, IL, USA) and GraphPad Prism. Chi-square test, Student's independent or paired *t* test, and Analysis of Variance (ANOVA) were used to assess the statistical significance for comparisons of the two groups. Pearson correlation analysis was used to analyze the correlations. Survival curves were analyzed with the Kaplan–Meier method and log-rank test. Univariate and multivariate analyses were conducted by a Cox proportional hazard regression model. *P* < 0.05 was considered statistical significance.

## Results

### Identification of a novel circDUSP16 in GC cells

**Fig. 1:**
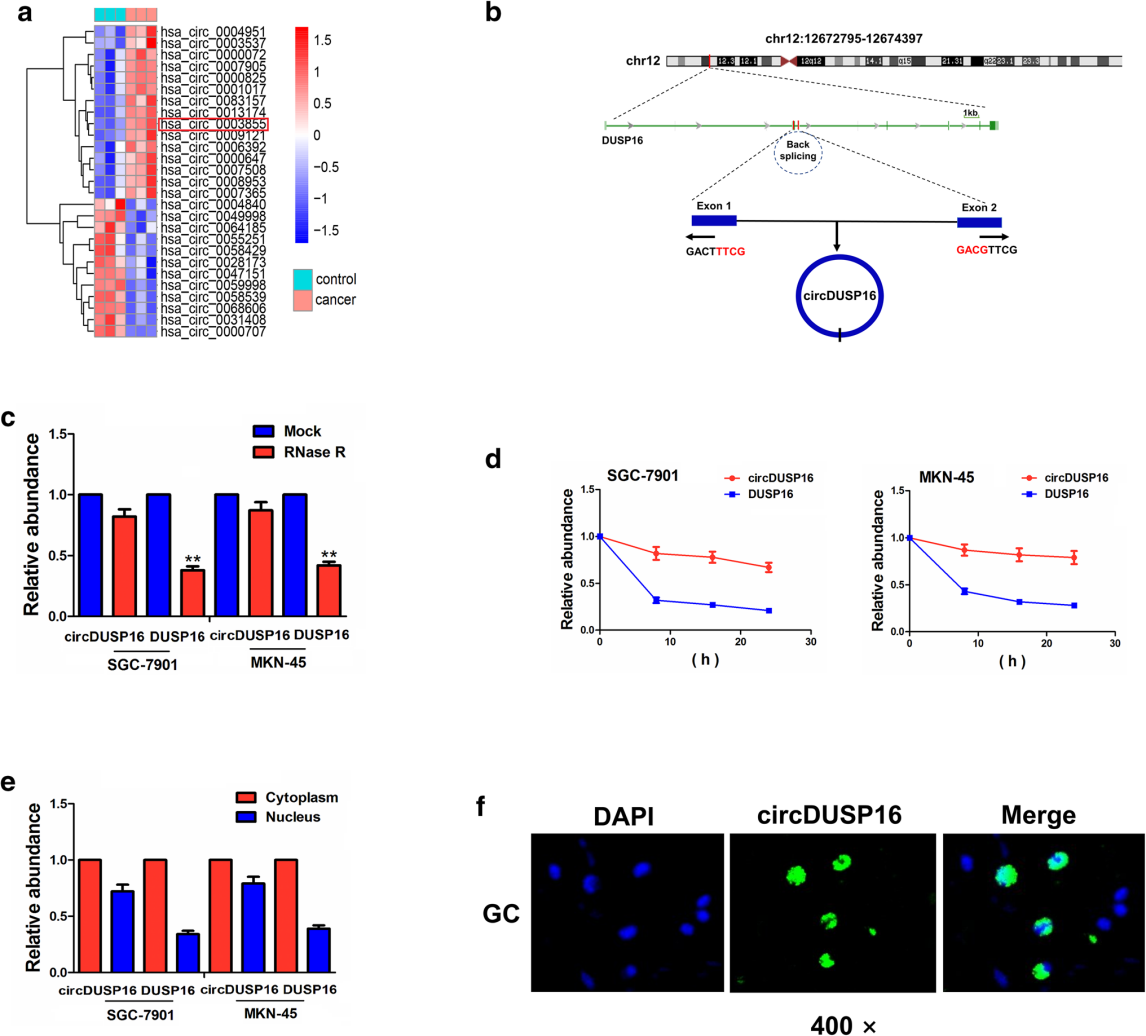
Identification of a novel circDUSP16 in GC cells. **a** GSE78092 analysis of the differentially expressed circRNAs between GC and adjacent normal tissues. **b** The genomic loci of the DUSP16 gene and circDUSP16. Arrows represent divergent primers that bind to the genomic region of circDUSP16. **c** qRT-PCR analysis of circDUSP16 and DUSP16 expression after treatment with RNase R in SGC-7901 and MKN-45 cells. **d** qRT-PCR analysis of the half-life of circDUSP16 and DUSP16 after treatment with Actinomycin D in SGC-7901 and MKN-45 cells. **e**, **f** qRT-PCR and FISH analysis of the cellular localization of circDUSP16 in GC cells and tissue cells. Data are the means ± SEM of 3 experiments. **P < 0.01

Based on the GEO database, a microarray chip GSE78092 was used to screen the differentially expressed circRNAs between GC and adjacent normal tissues (*n* = 3). With the criteria of FC > 2 and *P* value < 0.01, 12 downregulated circRNAs and 15 upregulated circRNAs were identified, of which hsa_circ_0003855 had a significantly increased expression in GC tissues (FC = 3.6, *P* = 0.0009; Fig. 1a).

We found that hsa_circ_0003855 (chr12:12,672,795-12,674,397) is derived from exon 1, 2 regions within dual specificity phosphatase 16 (DUSP16) locus, and is named as circDUSP16 (Fig. 1b). When compared with the linear DUSP16, circDUSP16 produced a substantial resistance to RNase R treatment in GC cell lines (SGC-7901 and MN-45), indicating that circDUSP16 harbored a loop structure in GC cells (Fig. 1c, ^**^P < 0.01, independent *t* test). We investigated the stability and cellular localization of circDUSP16 in GC cells. After SGC-7901 and MN-45 cells were treated with the transcription inhibitor Actinomycin D, the half-life of circDUSP16 could arrive at 24 h, but that of DUSP16 was less than 8 h in these two cells (Fig. 1d). The qRT–PCR and FISH analysis showed that circDUSP16 was predominantly localized in the cytoplasm in GC cell lines (Fig. 1e) and tissue cells (Fig. 1f).

### Upregulation of circDUSP16 was associated with poor survival in GC patients

**Fig. 2:**
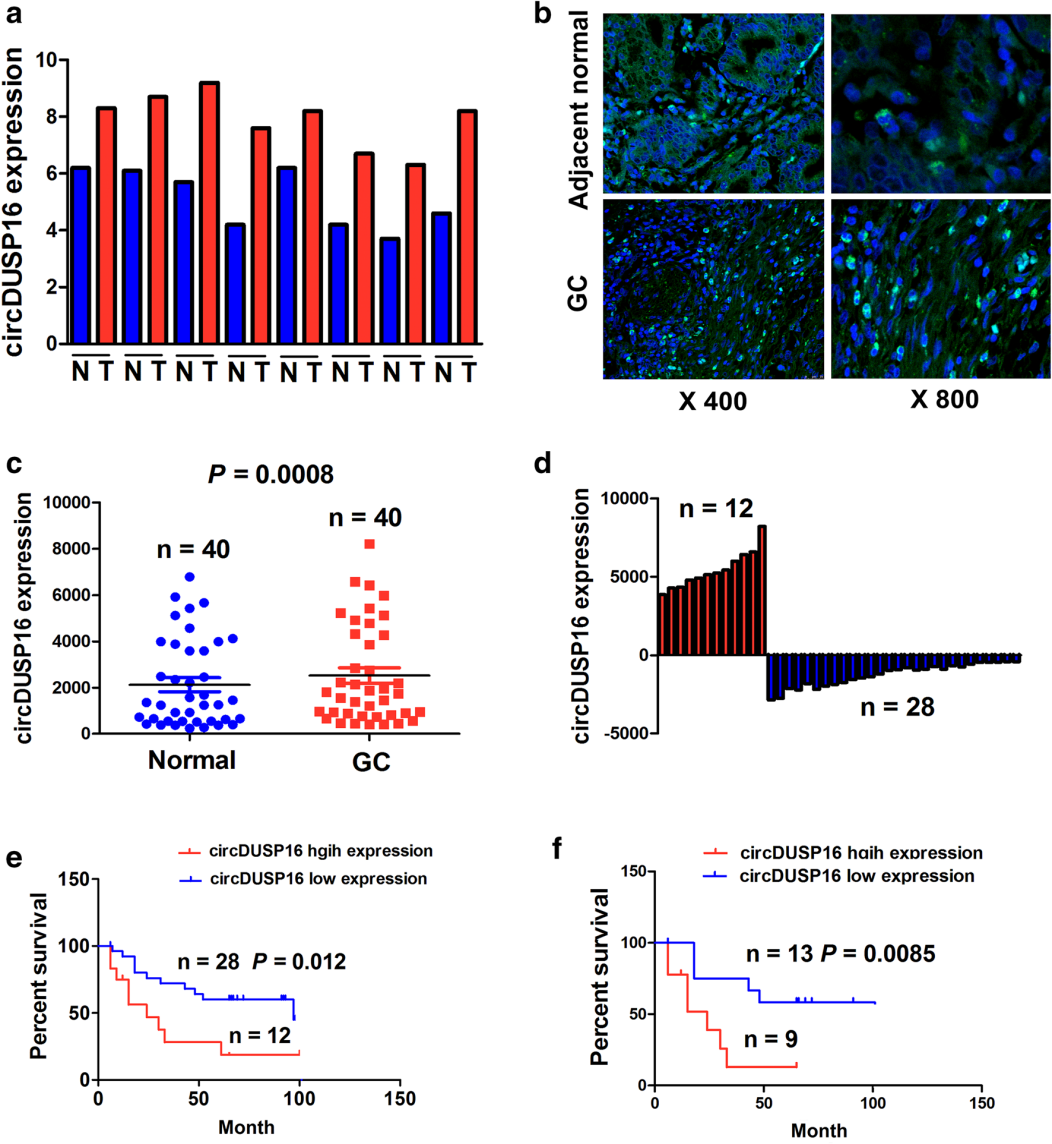
Upregulation of circDUSP16 was associated with poor survival in GC patients. **a** qRT–PCR analysis of the expressed levels of circDUSP16 in eight paired GC tissues. **b**, **c** FISH analysis of the expression levels of circDUSP16 in 40 paired GC tissues. **d** The cut-off value of circDUSP16 divided the GC patients into high expression (*n* = 12) and low expression groups (*n* = 28). **e**, **f** Kaplan–Meier analysis of the association of high or low circDUSP16 expression with overall survival in GC patients or those in early stage

The expression of circDUSP16 was found increased in GC tissues as compared with adjacent normal tissues, indicated by qRT–PCR analysis (*n* = 8; Fig. 2a). Then, this result was validated in 40 paired GC tissues by FISH analysis (*P* = 0.0008; Fig. 2b, c; paired *t* test). According to the survival time, survival status, and circDUSP16 expression, a cut-off value (3800) of circDUSP16 was obtained in GC, and divided the patients into high circDUSP16 expression (*n* = 12) and low circDUSP16 expression groups (*n* = 28; Fig. 2d).

We found that high expression of circDUSP16 had no association with the clinicopathological factors in GC patients (*P* > 0.05, Supplementary Table S2). Kaplan–Meier analysis demonstrated that the patients with high circDUSP16 expression possessed a shorter survival as compared with those with low circDUSP16 expression (*P* = 0.012, Fig. 2e). Based on TNM stage, the early stage patients rather than the late-stage ones (Supplementary Fig. S1) with high circDUSP16 expression showed a shorter survival as compared with those with low circDUSP16 expression (Fig. 2f). Univariate and multivariate analyses unveiled that high expression of circDUSP16 was an independent prognostic factor of poor survival in patients with GC (Supplementary Table S3).

### Knockdown of circDUSP16 inhibited the growth and invasion of GC cells

**Fig. 3:**
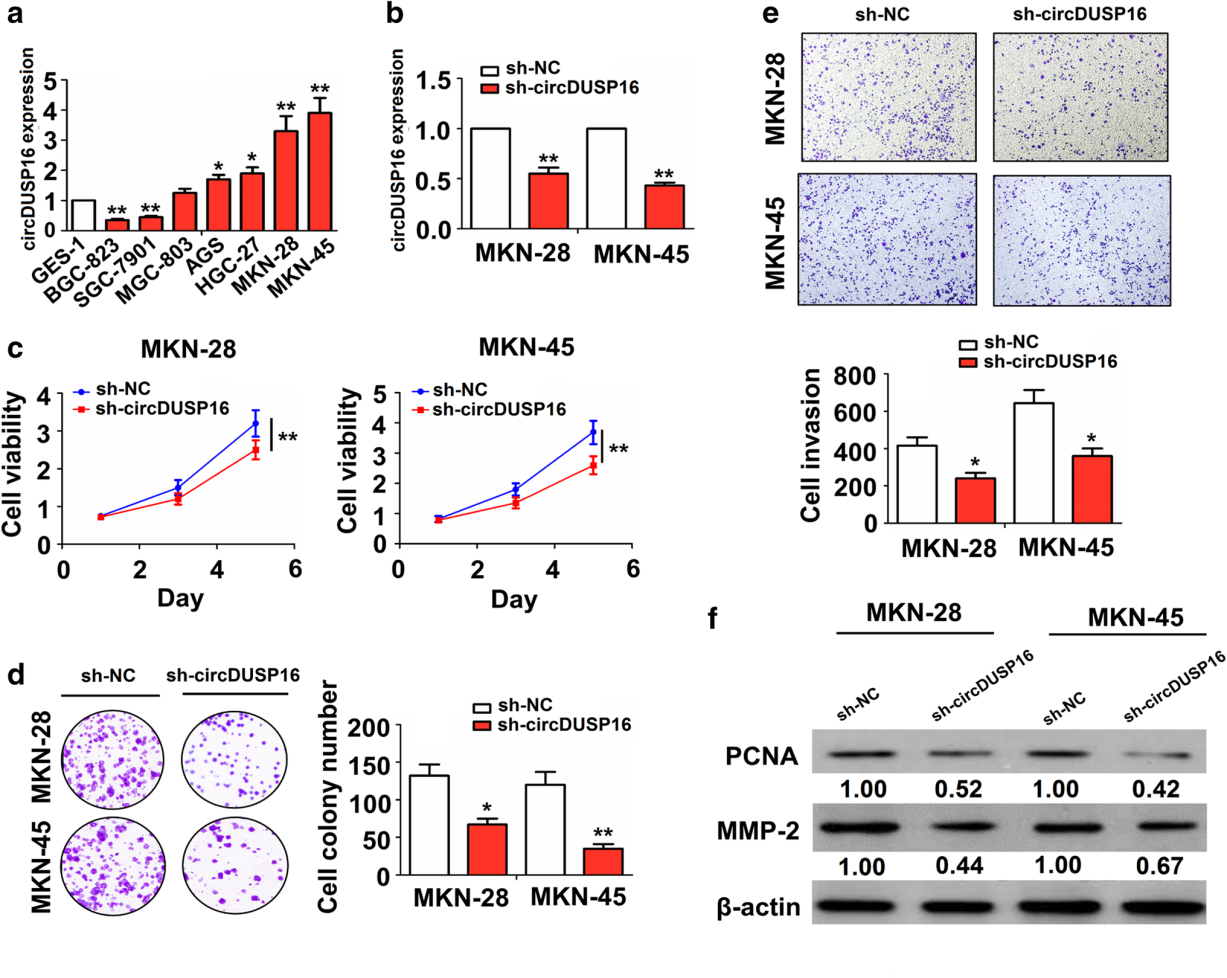
Knockdown of circDUSP16 inhibited cell proliferation, colony formation, and invasion in GC cells. **a** qRT–PCR analysis of the expression levels of circDUSP16 in different GC cell lines. **b** qRT–PCR analysis of the transfection efficiency of sh-circDUSP16 in MKN-28 and MKN-45 cell lines. **c** MTT, **d** colony formation, and **e** transwell analysis of the cell viability, colony number, and cell invasion after the transfection with sh-circDUSP16 in MKN-28 and MKN-45 cell lines. **f** Western blot analysis of the protein expression of PCNA and MMP2 after the transfection with sh-circDUSP16 in MKN-28 and MKN-45 cell lines. Data are the means ± SEM of 3 experiments. *P < 0.05, **P < 0.01

We constructed shRNA sequences against the back-splicing site of circDUSP16. The expression of circDUSP16 was examined in different GC cell lines by qRT–PCR analysis, indicating that circDUSP16 had a higher expression in MKN-28 and MKN-45 cell lines, but a lower expression in BGC-823 and SGC-7901 cell lines (Fig. 3a; ^*^P < 0.05, ^**^P < 0.01, independent *t* test). Then, the transfection efficiency of sh-circDUSP16 in MKN-28 and MKN-45 cell lines was determined by qRT–PCR analysis (Fig. 3b; ^**^P < 0.01, independent *t* test). We found that the cell viability (Fig. 3c; ^**^P < 0.01, independent *t* test), colony formation number (Fig. 3d; ^*^P < 0.05, ^**^P < 0.01, independent *t* test), and cell invasive potential (Fig. 3e; ^*^P < 0.05, independent *t* test) were decreased by the transfection with sh-circDUSP16 in MKN-28 and MKN-45 cell lines.

It was reported that proliferating cell nuclear antigen (PCNA) is closely related to DNA synthesis and acts a key role in the initiation of cell proliferation. It is a good indicator of cell proliferation in GC [24]. Matrix metalloproteinase 2 (MMP2) can degrade extracellular matrix proteins and participate in cell invasion-related signal transduction in GC [25]. We found that the protein expression of PCNA and MMP2, indicated by Western blot analysis, was downregulated by knockdown of circDUSP16 in MKN-28 and MKN-45 cell lines as compared with sh-NC group (Fig. 3f).

### Restored expression of circDUSP16 promoted the growth and invasion of GC cells

**Fig. 4:**
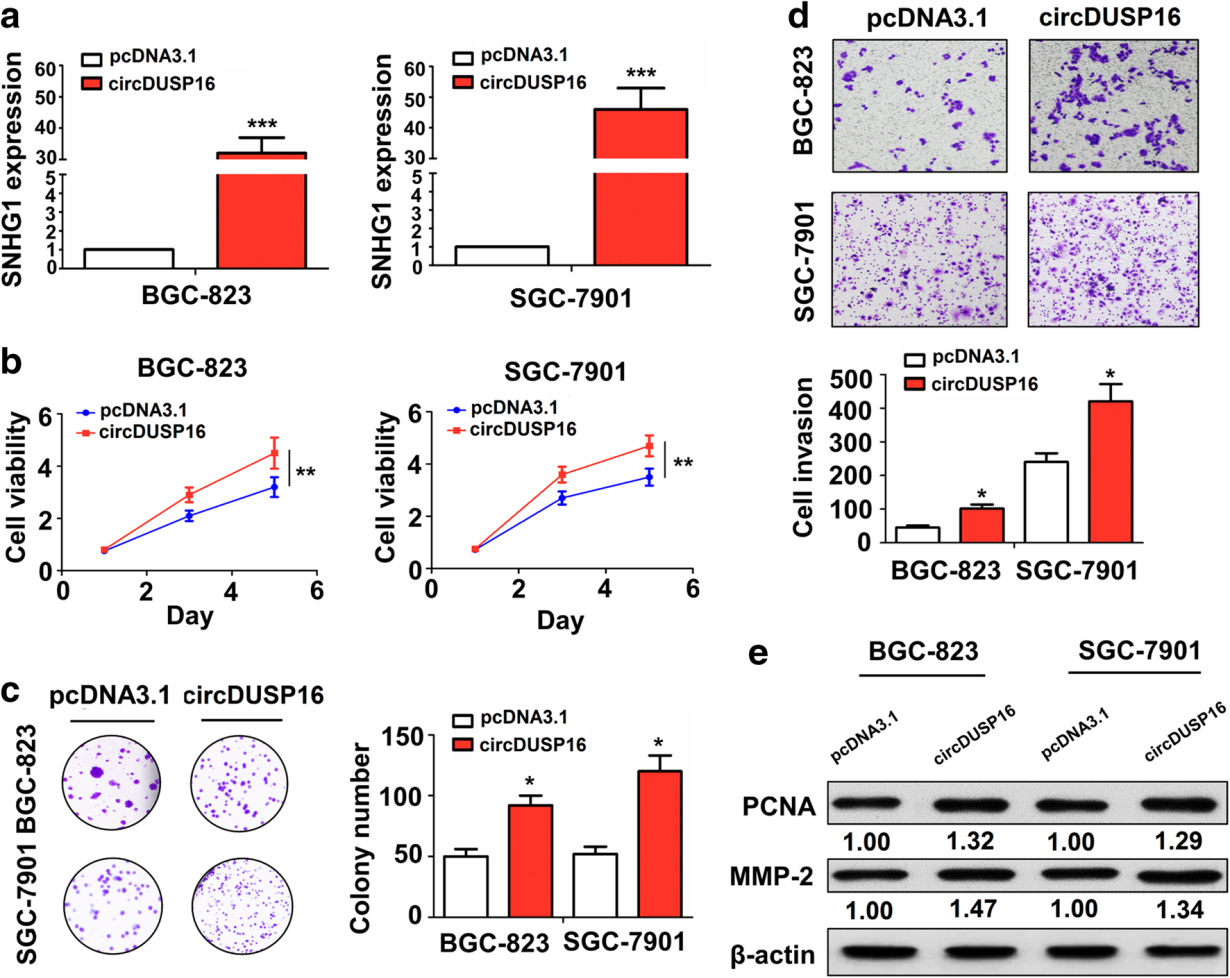
Overexpression of circDUSP16 facilitated cell proliferation, colony formation, and invasion in GC cells. **a** qRT–PCR analysis of the transfection efficiency of circDUSP16 plasmid in BGC-823 and SGC-7901 cell lines. **b** MTT, **c** colony formation, and **d** transwell analysis of the cell viability, colony number, and cell invasion after the transfection with circDUSP16 plasmid in BGC-823 and SGC-7901 cell lines. **e** Western blot analysis of the protein expression of PCNA and MMP2 after the transfection with circDUSP16 plasmid in BGC-823 and SGC-7901 cell lines. Data are the means ± SEM of 3 experiments. *P < 0.05, **P < 0.01, ***P < 0.001

The transfection efficiency of circDUSP16 plasmid in BGC-823 and SGC-7901 cell lines was assessed by qRT-PCR analysis (Fig. 4a; ^***^P < 0.001, independent *t* test). We found that the cell viability (Fig. 4b; ^**^P < 0.01, independent *t* test), colony formation number (Fig. 4c; ^*^P < 0.05, independent *t* test), and cell invasive potential (Fig. 4d; ^*^P < 0.05, independent *t* test) were enhanced by the transfection with circDUSP16 plasmid in BGC-823 and SGC-7901 cell lines. Western blot analysis indicated that the protein expression of PCNA and MMP2 was upregulated by circDUSP16 overexpression in BGC-823 and SGC-7901 cell lines as compared with the pcDNA3.1 group (Fig. 4e).

### circDUSP16 acted as a sponge of miR-145-5p in GC cells

**Fig. 5:**
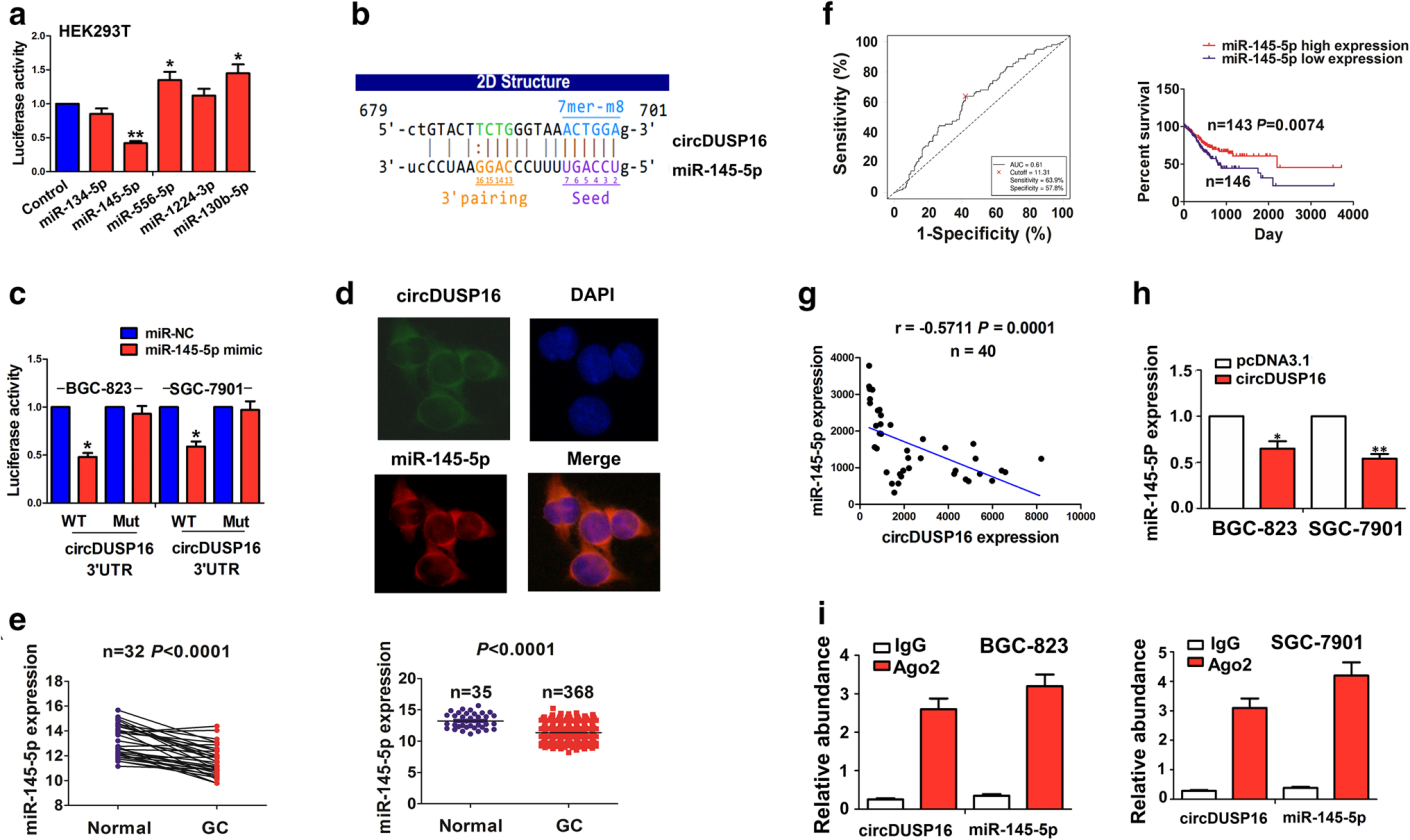
CircDUSP16 acted as a sponge of miR-145-5p in GC cells. **a** Luciferase activity of WT circDUSP16 3′UTR after the transfection with miR-134-5p, miR-145-5p, miR-556-5p, miR-1224-3p, and miR-130b-5p in HEK293T cells. **b** Schematic representation of the binding sites of miR-145-5p with circDUSP16. **c** Luciferase activity of WT or Mut circDUSP16 3′UTR after the transfection with miR-145-5p mimic or miR-NC in BGC-823 and SGC-7901 cell lines. **d** FISH analysis of the co-localization of circDUSP16 with miR-145-5p in BGC-823 cells. **e** TCGA analysis of the expression of miR-145-5p in paired (*n* = 32) and unpaired GC tissue samples (*n* = 368). **f** ROC curve analysis of the cut-off value of miR-145-5p in GC patients and Kaplan–Meier analysis of the association of high or low miR-145-5p expression with overall survival in GC patients. **g** Pearson correlation analysis of the correlation of circDUSP16 expression with miR-145-5p in GC tissues. **h** qRT–PCR analysis of the expression levels of miR-145-5p after the transfection with circDUSP16 plasmid in BGC-823 and SGC-7901 cell lines. **i** RIP analysis of the enrichment of circDUSP16 and miR-145-5p pulled down from the Ago2 protein in BGC-823 and SGC-7901 cell lines. Data are the means ± SEM of 3 experiments. *P < 0.05, **P < 0.01

We identified circDUSP16-specific binding with miRNAs including miR-134-5p, miR-145-5p, miR-556-5p, miR-1224-3p, and miR-130b-5p using the circular RNA interactome, and found that the luciferase activity of circDUSP16 3′UTR was significantly reduced by miR-145-5p as compared with the other miRNAs in HEK293T cells (Fig. 5a; ^*^P < 0.05, ^**^P < 0.01, independent *t* test). The binding sites of miR-145-5p with circDUSP16 3′UTR are indicated in Fig. 5b. We co-transfected miR-145-5p mimic and WT or Mut circDUSP16 3′UTR into BGC-823 and SGC-7901 cells, and found that miR-145-5p lowered the luciferase activity of WT circDUSP16 3′UTR, but had no effects on that of Mut circDUSP16 3′UTR as compared with the miR-NC group (Fig. 5c, ^*^P < 0.05, independent t test). FISH analysis showed that circDUSP16 was co-localized with miR-145-5p in the cytoplasm of BGC-823 cells (Fig. 5d).

We then found that miR-145-5p expression was downregulated in paired (*n* = 32, *P* < 0.0001, paired *t* test) and unpaired GC tissue samples (Fig. 5e; *n* = 368, *P* < 0.0001, independent *t* test). The cut-off value (11.31) of miR-145-5p divided the patients into high miR-145-5p expression (*n* = 143) and low miR-145-5p expression groups (*n* = 146), and the patients with low miR-145-5p expression had a poorer survival as compared with those with high miR-145-5p expression (*P* = 0.0074; Fig. 5f). Based on the TNM stage, both of early stage and late-stage patients with low miR-145-5p expression showed a poorer survival as compared with those with high miR-145-5p expression (Supplementary Fig. S2). In addition, high expression of miR-145-5p had no association with the clinicopathological parameters (*P* > 0.05; Supplementary Table S4), but acted as an independent prognostic factor of poor survival in patients with GC (*P* < 0.0001; Supplementary Table S5).

Pearson correlation analysis indicated that circDUSP16 had a negative correlation with miR-145-5p expression in GC tissue samples (*r* = − 0.5711, *P* = 0.0001; Fig. 5g). The qRT-PCR analysis showed that overexpression of circDUSP16 reduced the expression levels of miR-145-5p (Fig. 5h; ^*^P < 0.05, ^**^P < 0.01, independent *t* test), but miR-145-5p had no effects on circDUSP15 expression in BGC-823 and SGC-7901 cells (Supplementary Fig. S3). RIP assay was executed for RNA pulled down from Ago2 protein in BGC-823 and SGC-7901 cells, and the endogenous expression of circDUSP16 and miR-145-5p was enriched in the Ago2 pellet as compared with the input control, indicated by qRT-PCR analysis (Fig. 5i).

### miR-145-5p reversed the tumor-promoting effects of circDUSP16 in GC cells

**Fig. 6:**
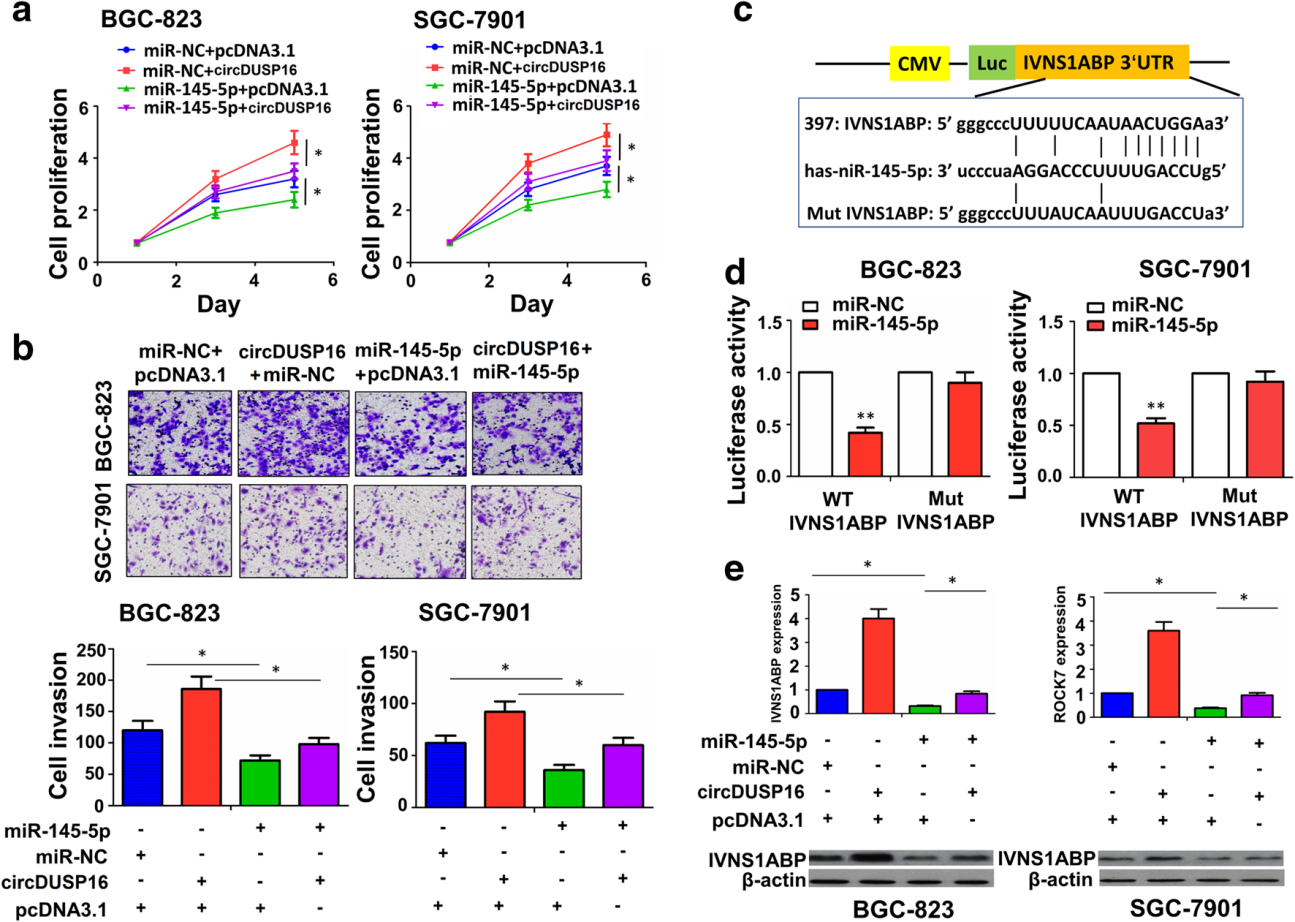
MiR-145-5p reversed the tumor-promoting effects of circDUSP16 in GC cells. **a** MTT and **b** transwell analysis of the effects of co-transfection with circDUSP16 and miR-145-5p mimic on cell proliferation and invasive potential in BGC-823 and SGC-7901 cell lines. **c** Schematic representation of the binding sites between miR-145-5p and WT or Mut IVNS1ABP 3′ UTR. **d** Luciferase activity of WT or Mut IVNS1ABP 3′ UTR after co-transfection with miR-145-5p mimic and WT or Mut IVNS1ABP 3′ UTR reporter in BGC-823 and SGC-7901 cell lines. **e** qRT-PCR and Western blot analysis of IVNS1ABP expression levels after co-transfection with circDUSP16 and miR-145-5p mimic in BGC-823 and SGC-7901 cells. Data shown are the mean ± SEM of three experiments. *P < 0.05; **P < 0.01

To uncover the functional interaction between circDUSP16 and miR-145-5p in GC cells, the miR-145-5p mimic and circDUSP16 plasmid were co-transfected into BGC-823 and SGC-7901 cell lines, indicating that as compared with the group miR-NC + pcDNA3.1 or miR-NC + circDUSP16, miR-145-5p inhibited the cell proliferation and invasion, and counteracted the tumor-promoting effects of circDUSP16 in these two cell lines (Fig. 6a, b; ^*^P < 0.05, ANOVA test).

We further identified 16 target genes of miR-145-5p using starBasev2.0 (Supplementary Fig. S4). The expression levels of these target genes (Supplementary Fig. S5) and their correlation with miR-145-5p expression were validated in GC tissues (Supplementary Fig. S6), of which IVNS1ABP exhibited a most significantly negative correlation with miR-145-5p expression in GC samples (*r* = − 0.3877, *P* < 0.0001; Supplementary Fig. S6). The binding sites of miR-145-5p with WT or Mut IVNS1ABP 3′ UTR are indicated in Fig. 6c. We co-transfected BGC-823 and SGC-7901 cells with WT or Mut IVNS1ABP 3′ UTR and miR-145-5p mimic, and found that the miR-145-5p decreased the luciferase activity of WT IVNS1ABP 3′ UTR, but exerted no effects on that of Mut IVNS1ABP 3′ UTR as compared with the miR-NC group (Fig. 6d; ^**^P < 0.01, independent *t* test). The qRT–PCR and Western blot analysis indicated that miR-145-5p decreased IVNS1ABP expression and reversed circDUSP16-induced IVNS1ABP expression in BGC-823 and SGC-7901 cells (Fig. 6e; ^*^P < 0.05, ANOVA test).

### Knockdown of circDUSP16 suppressed in vivo tumor growth

**Fig. 7:**
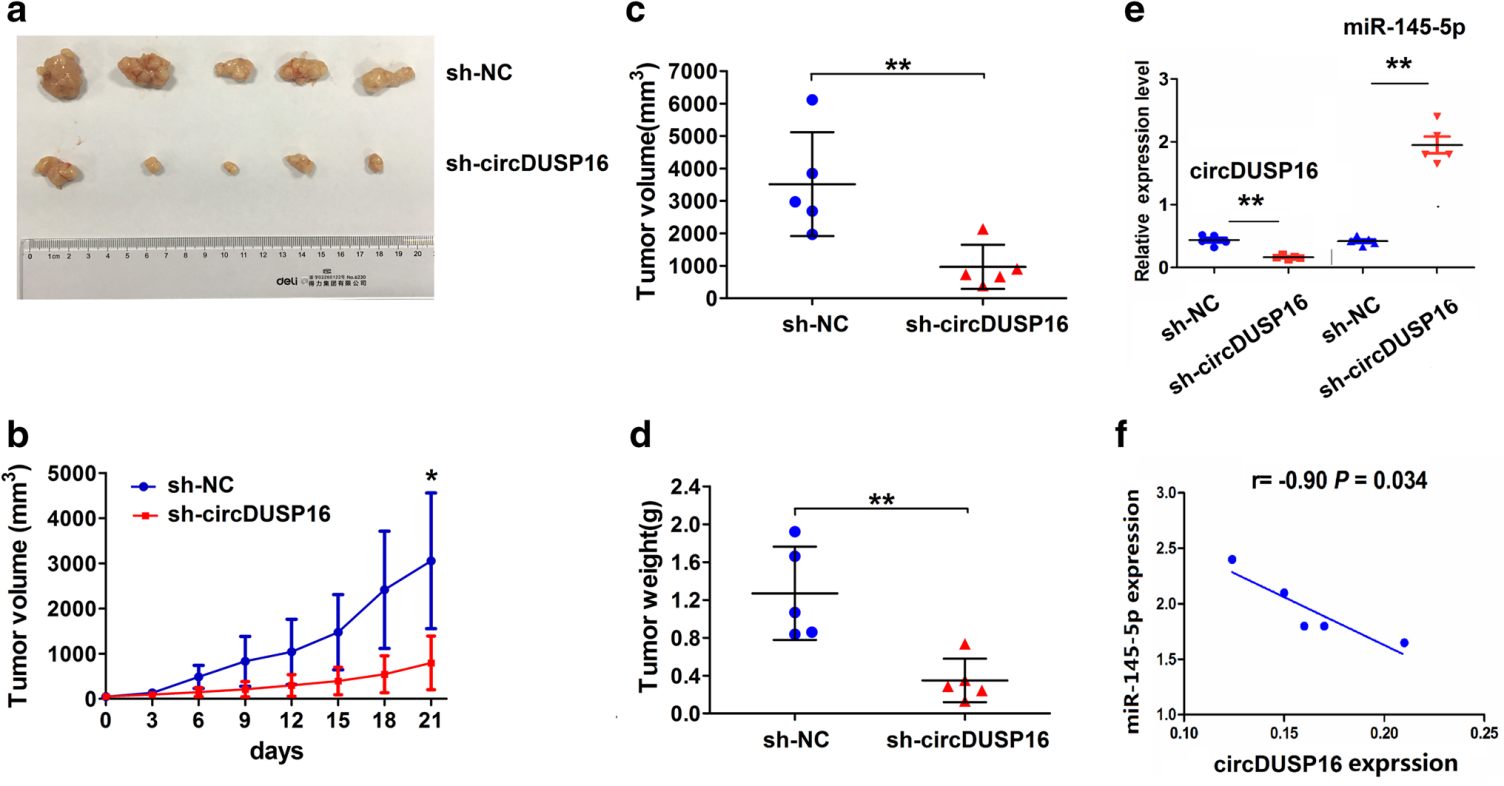
Knockdown of circDUSP16 inhibited in vivo tumor growth. **a** Representative photographs of MKN-45 xenograft tumors after treatment with sh-circDUSP16 or sh-NC. **b** Growth curve analysis of tumor proliferation activity after treatment with sh-circDUSP16 or sh-NC. **c**, **d** Comparison of the tumor volumes and weights between the sh-circDUSP16 and sh-NC groups. **e** qRT-PCR analysis of circDUSP16 and miR-145-5p expression levels after treatment with sh-circDUSP16 or sh-NC. **f** Pearson correlation analysis of the relationship between circDUSP16 and miR-145-5p expression in sh-circDUSP16-treated tumor tissues. **P < 0.01

To confirm the effects of circDUSP16 on in vivo tumor growth, a xenograft tumor model was established to observe the tumor growth after subcutaneous inoculation with sh-circDUSP16 stably transfected MKN-45 cells. During a tumor growth period, the length and weight of xenograft tumors were detected. We found that the growth ability of the xenograft tumors was reduced in sh-circDUSP16 group as compared with the sh-NC group (Fig. 7a, b; ^*^P < 0.05, independent *t* test). After the tumor tissues were harvested, the average volumes and weights were lowered in sh-circDUSP16 group as compared with the sh-NC group (Fig. 7c, d; ^**^P < 0.01, independent *t* test). The qRT-PCR analysis showed that circDUSP16 expression was decreased, but miR-145-5p expression was increased in sh-circDUSP16 group as compared with the sh-NC group (Fig. 7e; ^**^P < 0.01, independent *t* test), and Pearson correlation analysis revealed a negative correlation of circDUSP16 with miR-145-5p expression in sh-circDUSP16-transfected tumor tissues (Fig. 7f, *P* = 0.034).

## Discussion

Increasing evidence indicated that circRNAs act as potential biomarkers in patients with GC. Upregulation of circ-DONSON, circAKT3, and circDLST [8, 11, 13] or downregulation of KIAA1244, circPSMC3, and circFAT1(e2) [14-16] is regarded as independent prognostic factors of poor prognosis in patients with GC. Herein, we identified a novel circDUSP16, and it was resistant to RNase R and localized in the cytoplasm of GC cells. The expression of circDUSP16 was increased in GC tissue samples and its high expression was an independent prognostic factor of poor survival in GC patients. These results indicated that circDUSP16 might be a potential biomarker for GC patients.

Functionally, circRNAs can act as oncogenes or tumor suppressors in GC. For one thing, circAKT3, circNRIP1, and circDLST facilitate tumor growth and metastasis and enhance cisplatin resistance in GC cells [11-13]. For another thing, circPSMC3 and circFAT1(e2) inhibit the tumorigenesis and invasion of GC cells [15, 16]. However, the functional role of circDUSP16 in GC remains unclear. Herein, we explored the role of circDUSP16 in GC cells, and found that knockdown of circDUSP16 repressed cell growth and invasion in vitro and in vivo, but overexpression of circDUSP16 reversed these effects. These studies indicated that circDUSP16 might be a tumor-promoting factor in GC cells.

Mechanistically, circRNA can sponge miRNA to participate in the progression of GC [11, 13]. Previous studies showed that circZNF609, circ_0058063, and circCEP128 sponge miR-145-5p to promote cancer progression [26-28]. Likewise, we identified that circDUSP16 was co-localized with miR-145-5p in the cytoplasm of GC cells, and displayed a negative correlation with miR-145-5p expression in GC tissue samples. Moreover, circDUSP16 reduced miR-145-5p expression levels and could bind with miR-145-5p/Ago2 complex in GC cells. These studies indicated that circDUSP16 acted as a sponge of miR-145-5p in GC cells.

It was reported that miR-145-5p expression is decreased in breast cancer and esophageal carcinoma, and its downregulation is associated with distal metastasis, tumor differentiation, and poor survival in GC patients [29-31]. miR-145-5p suppresses the proliferation and invasion by targeting SOX2 or SP1 [29, 31], and promotes GC differentiation by targeting KLF5 [30]. Herein, we found that miR-145-5p expression was downregulated in GC tissue samples, and its low expression was an independent prognostic factor of poor survival in GC patients. miR-145-5p also inhibited cell growth and invasion and attenuated circDUSP16-induced tumor-promoting effects in GC cells. It was shown that NS1-Binding Protein (NS1-BP) is implicated in c-Myc transcriptional control [32] and facilitates the radio-sensitivity by inhibiting c-Myc in esophageal squamous cell carcinoma [33]. We found that influenza virus NS1A-binding protein (IVNS1ABP) was a direct target of miR-145-5p and miR-145-5p reduced circDUSP16-induced IVNS1ABP expression in GC cells.

Taken together, our findings demonstrated that circSUSP16 acted as an independent prognostic factor of poor survival in GC patients, and promoted the tumorigenesis and invasion of GC cells by sponging miR-145-5p/ IVNS1ABP axis.

## Electronic supplementary material

Below is the link to the electronic supplementary material.

Supplementary file1 (DOC 123 kb)

Supplementary file2 (PDF 634 kb)

Supplementary file3 (PDF 952 kb)

Supplementary file4 (PDF 807 kb)

Supplementary file5 (PDF 730 kb)

Supplementary file6 (PDF 1760 kb)

Supplementary file7 (PDF 1811 kb)
