# Supplementary material for: CircDUSP16 promotes the tumorigenesis and invasion of gastric cancer by sponging miR-145-5p
Source: Gastric Cancer. 2019 Nov 27;23(3):437–48. doi: 10.1007/s10120-019-01018-7 (PMC7165161; doi:10.1007/s10120-019-01018-7)
Supplement: Supplementary file 1 — Supplementary file1 (DOC 123 kb) [file 10120_2019_1018_MOESM1_ESM.doc]

**Supplementary Figure Legends**

**Supplementary Figure S1.** Kaplan-Meier analysis of the association of high or low circDUSP16 expression with overall survival in late-stage patients.

**Supplementary Figure S2.** Kaplan-Meier analysis of the association of high or low miR-145-5p expression with overall survival in early- or late-stage patients.

**Supplementary Figure S3.** RT-PCR analysis of the effects of miR-145-5p on circDUSP16 expression levels in BGC-823 and SGC-7901 cells. Data are the means ± SEM of 3 experiments. ***P* < 0.01.

**Supplementary Figure S4.** 16 target genes of miR-145-5p were identified by using starBasev2.0 prediction tool.

**Supplementary Figure S5.** TCGA analysis of the expression levels of miR-145-5p target genes in pared and unpaired GC tissue samples.

**Supplementary Figure S6.** Pearson correlation analysis of the correlation of miR-145-5p with its target gene expression in GC tissue samples.

**Supplementary Tables**

**Table S1** The primer sequences

| **Gene name** | **Primer sequence** | Annealing temperature (℃) | Product length (bp) |
| --- | --- | --- | --- |
| β-actin | F:5' GTGGCCGAGGACTTTGATTG3'  R:5’CCTGTAACAACGCATCTCATATT3’ | 60 | 73 |
| circDUSP16 | F:5’ CCCAAGATGTTGCCTCTCTC 3’  R:5’ AGCCAGCGCATTACATCATT 3’ | 60 | 72 |
| DUSP16 | F:5’ GCCCATGAGATGATTGGAACTC 3’  R:5’ CGGCTATCAATTAGCAGCACTTT 3’ | 60 | 72 |
| miR-145-5p | F: 5’CAGTCTTGTCCAGTTTTCCCAG3’ |  |  |
|  | R: 5’TATGCTTGTTCTCGTCTCTGTGTC3’ | 60 | 72 |
| U6 | F: 5’CCCTGGCACCCAGCAC3’ |  |  |
|  | R: 5’GCCGATCCACACGGAGTAC3’ | 60 | 72 |
| IVNS1ABP | F: 5’ TAATCAACTGGGTGCAGCGT3’  R: 5’ TATGGCCATTCTCACGTGGT3’ | 60 | 93 |

**Table S2** The association of circDUSP16 expression with clinicopathological

features in GC patients

| Parameters | Cases  (n) | circDUSP16 expression | | | *P* |
| --- | --- | --- | --- | --- | --- |
|  | 40 | Low  28 | High  12 |  | |
| ***Age*** |  |  |  |  | |
| ≥60 | 20 | 14 | 6 |  | |
| <60 | 20 | 14 | 6 | 1.000 | |
| ***Gender*** |  |  |  |  | |
| Female | 14 | 9 | 5 |  | |
| Male | 26 | 19 | 7 | 0.568 | |
| ***Tumor size (cm)*** |  |  |  |  | |
| <3.5 | 16 | 12 | 4 |  | |
| ≥3.5 | 24 | 16 | 8 | 0.578 | |
| ***Pathological stage*** |  |  |  |  | |
| I+II | 15 | 11 | 4 |  | |
| III | 25 | 17 | 8 | 0.726 | |
| ***TNM staging*** |  |  |  |  | |
| 1+2 | 18 | 15 | 3 |  | |
| 3+4 | 22 | 13 | 9 | 0.100 | |
| ***Lymph node metastasis*** |  |  |  |  | |
| Negative | 13 | 11 | 2 |  | |
| Positive | 27 | 17 | 10 | 0.167 | |

**Table S3** Univariate and multivariate Cox regression analysis of

the association of circDUSP16 with overall survival in GC patients

| Parameter | Univariate *P* |  | Multivariate analysis | | |
| --- | --- | --- | --- | --- | --- |
|  |  |  | *P* | HR | 95%CI |
| Age (≥60 vs. <60 years) | 0.713 |  | NA |  |  |
| Gender (Male vs. Female) | 0.109 |  | NA |  |  |
| Tumor size (≥3.5 vs. <3.5 cm) | 0.409 |  | NA |  |  |
| Pathological staging (III vs. I/II) | 0.066 |  | 0.040 | 2.988 | 1.053-8.364 |
| TNM staging (III/ vs. I/II) | 0.379 |  | NA |  |  |
| Lymph node metastasis (Positive vs. Negative) | 0.079 |  | 0.043 | 3.216 | 1.039-9.955 |
| circDUSP16 expression (High vs. low) | 0.018 |  | 0.038 | 2.629 | 1.053-8.364 |

NA: not analyzed

**Table S4** The correlation of miR-145-5p expression with clinicopathological

characteristics of GC patients

| Variables | Cases  (n) | miR-145-5p | | *P* value |
| --- | --- | --- | --- | --- |
|  |  | High | Low |  |
| Total | 289 | 143 | 146 |  |
| *Age (years)* |  |  |  |  |
| ≥60 | 197 | 93 | 104 |  |
| <60 | 92 | 50 | 42 | 0.312 |
| *Gender* |  |  |  |  |
| Male | 183 | 94 | 89 |  |
| Female | 106 | 50 | 56 | 0.542 |
| *Pathological stage* |  |  |  |  |
| Ⅰ/Ⅱ | 140 | 70 | 70 |  |
| Ⅲ/Ⅳ | 149 | 73 | 76 | 0.907 |
| *T stage* |  |  |  |  |
| T1/T2 | 73 | 37 | 36 |  |
| T3/T4 | 216 | 106 | 110 | 0.892 |
| *N stage* |  |  |  |  |
| Negative | 95 | 45 | 50 |  |
| Positive | 194 | 98 | 96 | 0.619 |
| *M stage* |  |  |  |  |
| Negative | 261 | 130 | 131 |  |
| Positive | 28 | 13 | 15 | 0.843 |

**Table S5 Cox regression analysis of miR-145-5p expression as survival predictor**

| Variables | Univariate Cox regression analysis | |  | | Multivariate Cox regression analysis | | |  |
| --- | --- | --- | --- | --- | --- | --- | --- | --- |
|  | RR (95% CI) | *P* value | |  | RR (95% CI) | *P* value | |  |
| *Age (years)* |  |  | |  |  | |  |  |
| ≥60 vs. <60 | 1.736 (1.097 to 2.747) | 0.018 | |  | 1.923 (1.214 to 3.048) | | 0.005 | |
| *Gender* |  |  | |  |  | |  | |
| Male vs. Female | 1.274 (0.831 to 1.953) | 0.267 | |  | NA | | NA | |
| *Pathological stage* |  |  | |  |  | |  | |
| Ⅲ/Ⅳ vs.Ⅰ/Ⅱ | 1.744 (1.148 to 2.648) | 0.009 | |  | 1.265 (0.739 to 2.167) | | 0.392 | |
| *T stage* |  |  | |  |  | |  | |
| T3+T4 vs. T1+T2 | 1.513 (0.913 to 2.507) | 0.108 | |  | NA | | NA | |
| *N staging* |  |  | |  |  | |  | |
| Positive vs. Negative | 2.023 (1.237 to 3.310) | 0.005 | |  | 1.847 (0.979 to 3.485) | | 0.058 | |
| *M stage* |  |  | |  |  | |  | |
| Positive vs. Negative | 1.571 (0.857 to 2.879) | 0.144 | |  | NA | | NA | |
| *miR-145-5p* |  |  | |  |  | |  | |
| High vs. Low | 2.265 (1.492 to 3.439) | <0.0001 | |  | 2.311 (1.520 to 3.514) | | <0.0001 | |

NA: not analyzed
