# Supplementary figures and images for: CircDUSP16 promotes the tumorigenesis and invasion of gastric cancer by sponging miR-145-5p
Source: Gastric Cancer. 2019 Nov 27;23(3):437–48. doi: 10.1007/s10120-019-01018-7 (PMC7165161; doi:10.1007/s10120-019-01018-7)

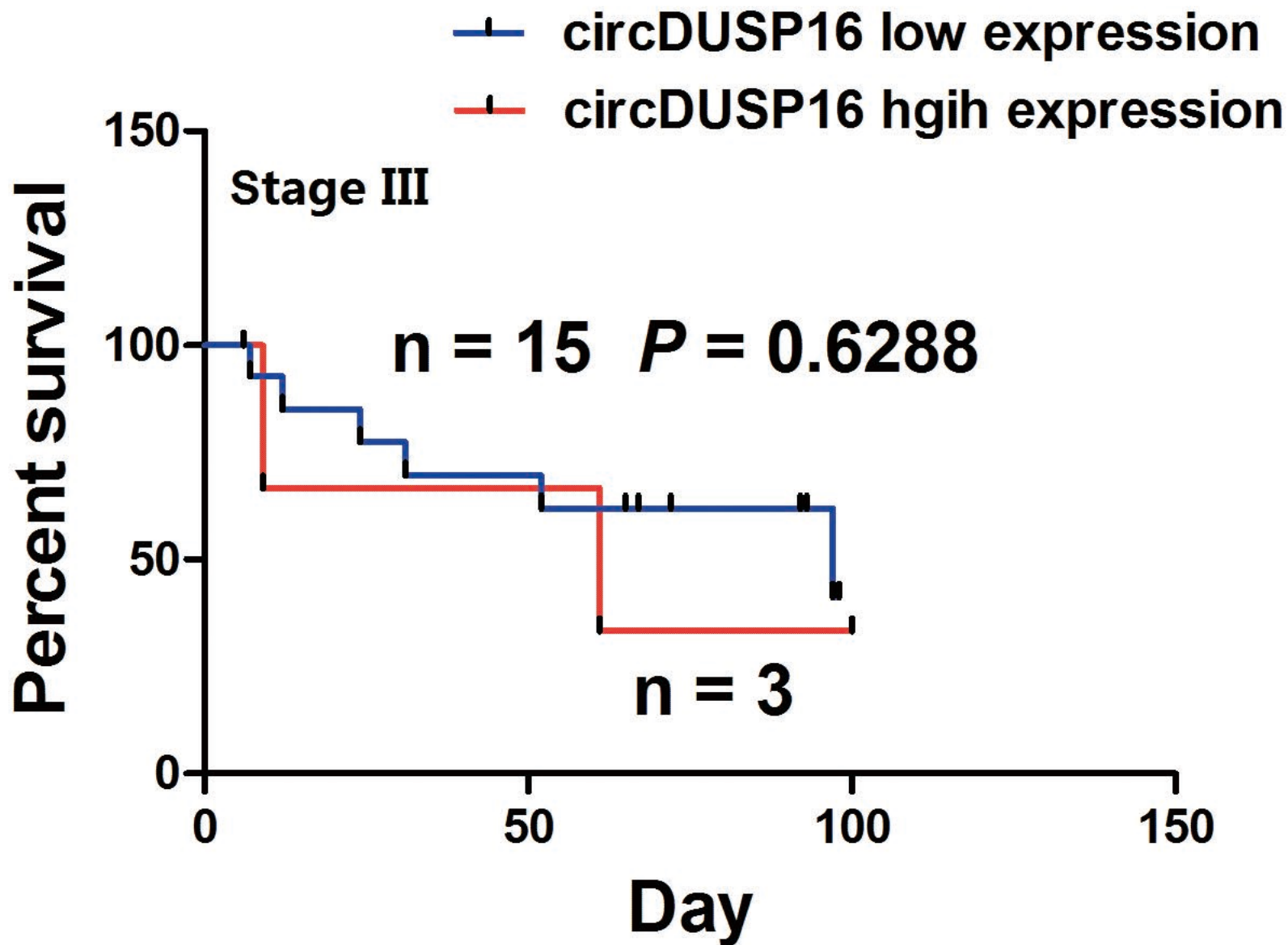

Supplement: Supplementary file 2 — Supplementary file2 (PDF 634 kb) [file 10120_2019_1018_MOESM2_ESM.pdf]

- miR-145-5p high expression
- miR-145-5p low expression

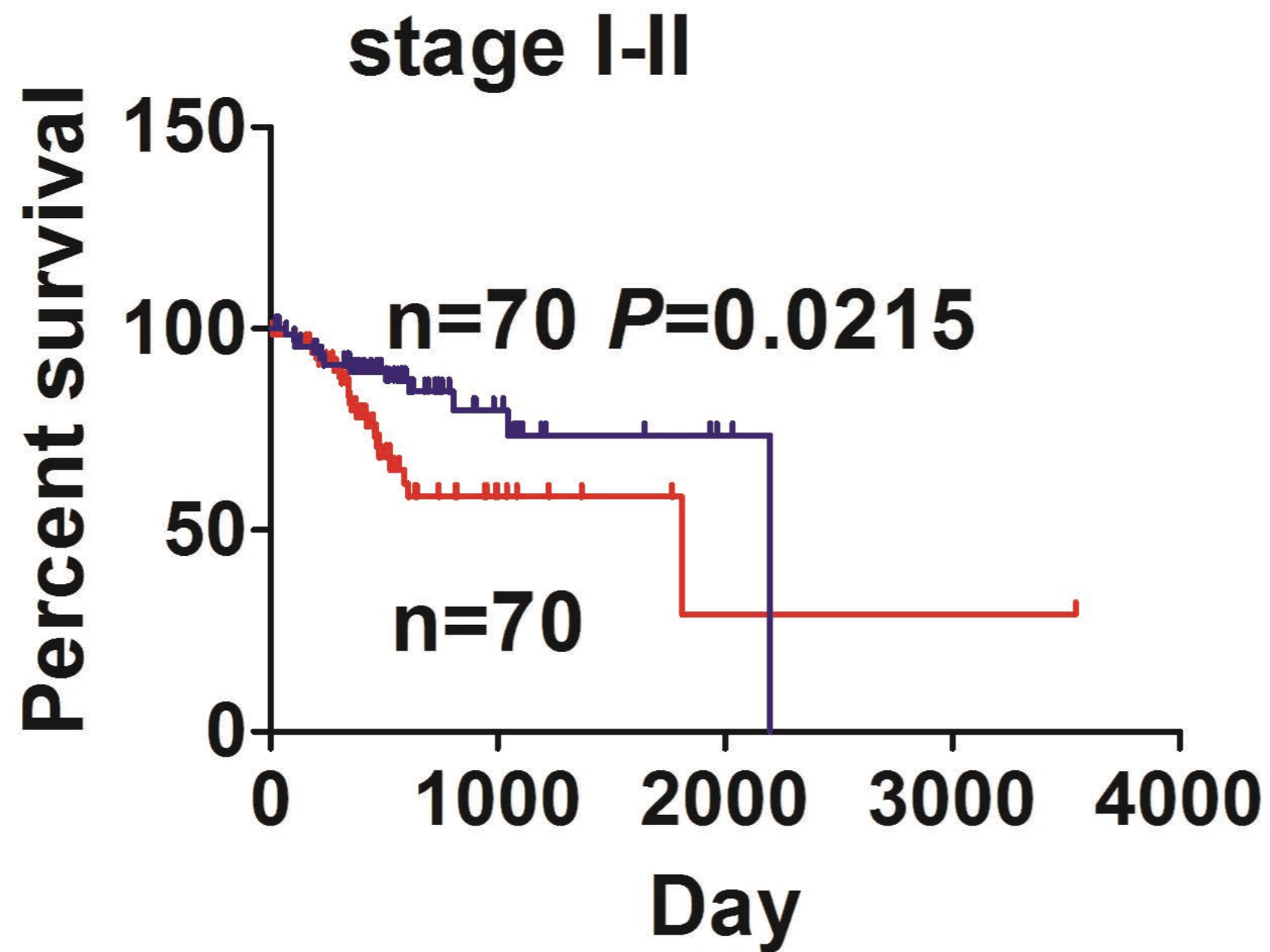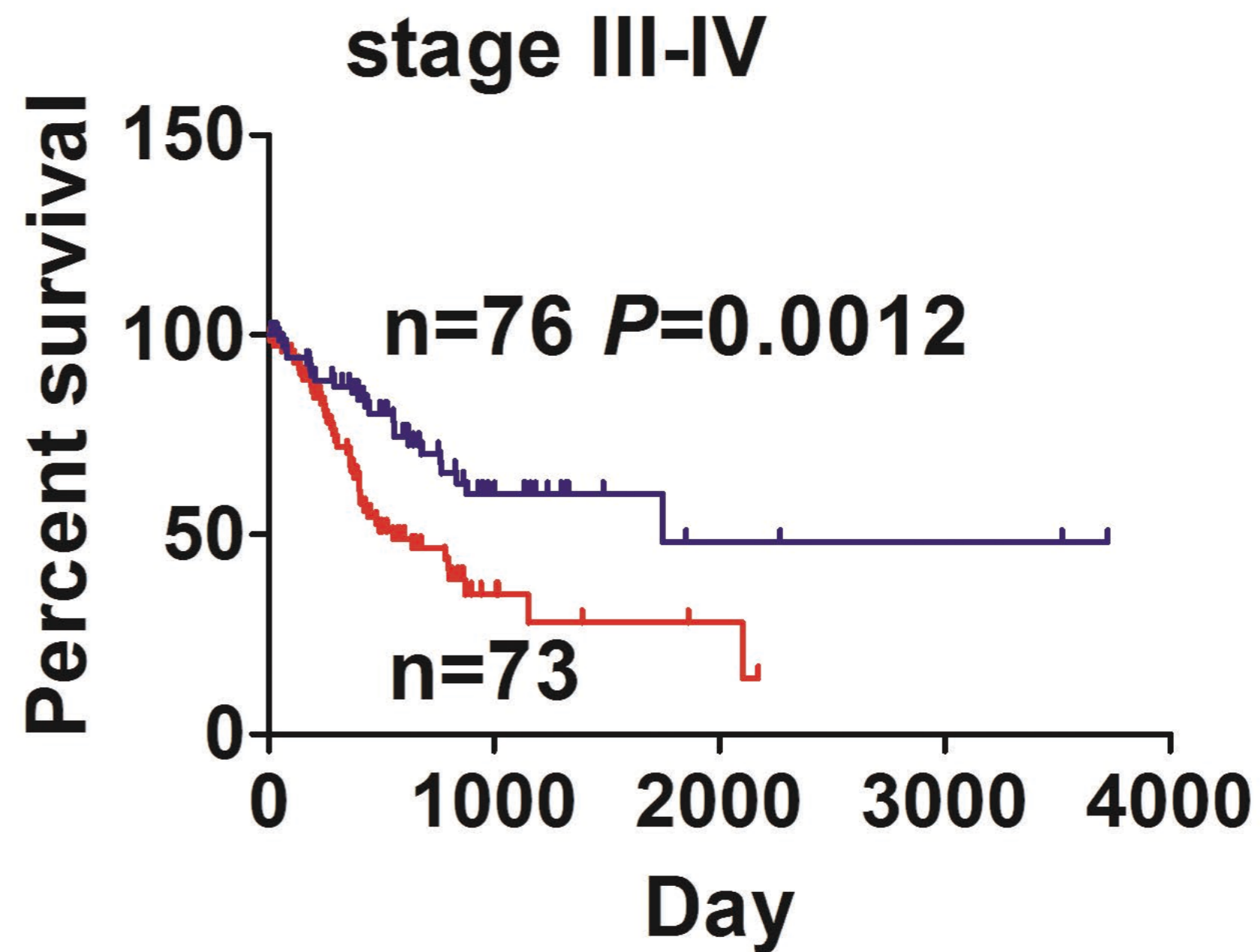

Supplement: Supplementary file 3 — Supplementary file3 (PDF 952 kb) [file 10120_2019_1018_MOESM3_ESM.pdf]

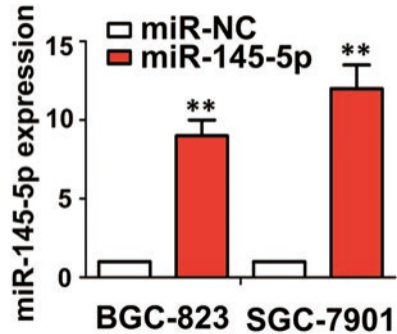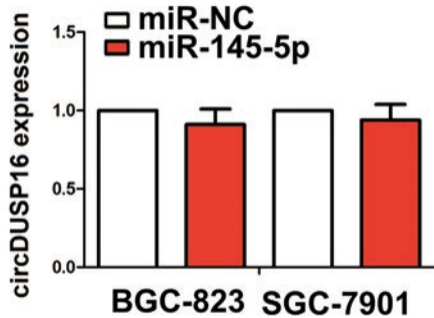

Supplement: Supplementary file 4 — Supplementary file4 (PDF 807 kb) [file 10120_2019_1018_MOESM4_ESM.pdf]

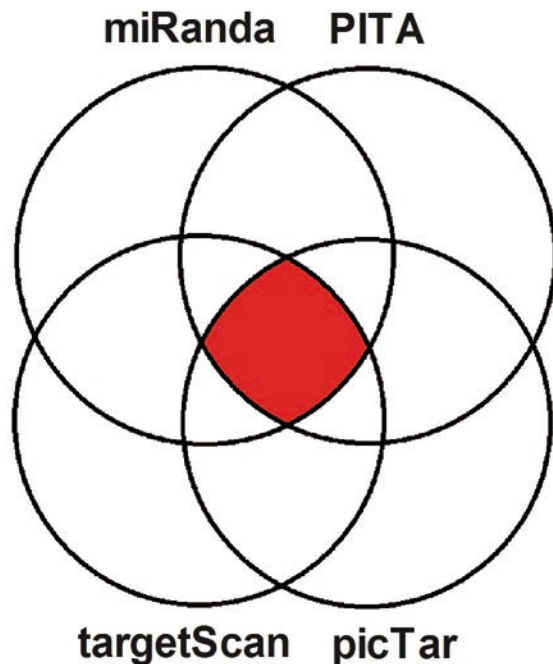

|   |          |        |
|---|----------|--------|
| ● | CCNL1    | ACTG1  |
|   | H2AFX    | BAZ2A  |
|   | TAGLN2   | SPATS2 |
|   | RTKN     | QSER1  |
|   | NUFIP2   | ARF6   |
|   | FAM135A  | EFNA3  |
|   | IVNS1ABP | FBXO28 |
|   | EIF4A2   | SOX11  |

Supplement: Supplementary file 5 — Supplementary file5 (PDF 730 kb) [file 10120_2019_1018_MOESM5_ESM.pdf]

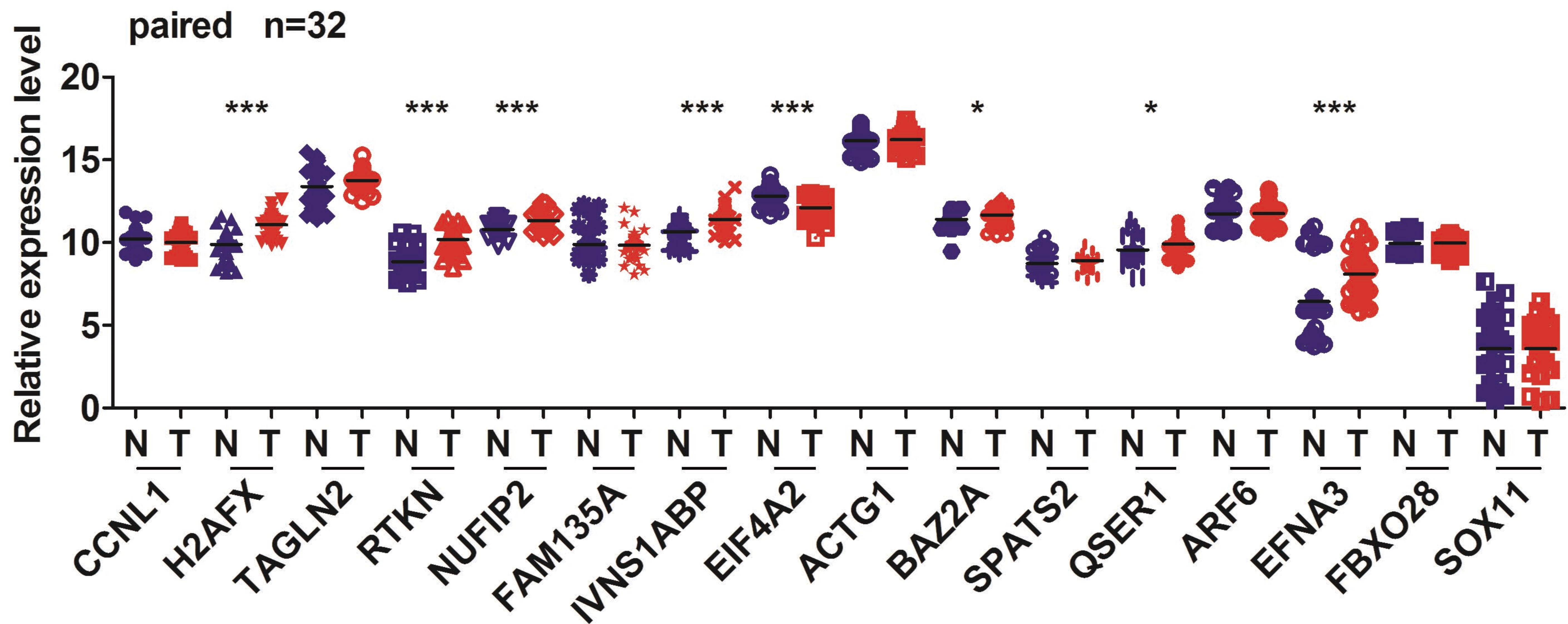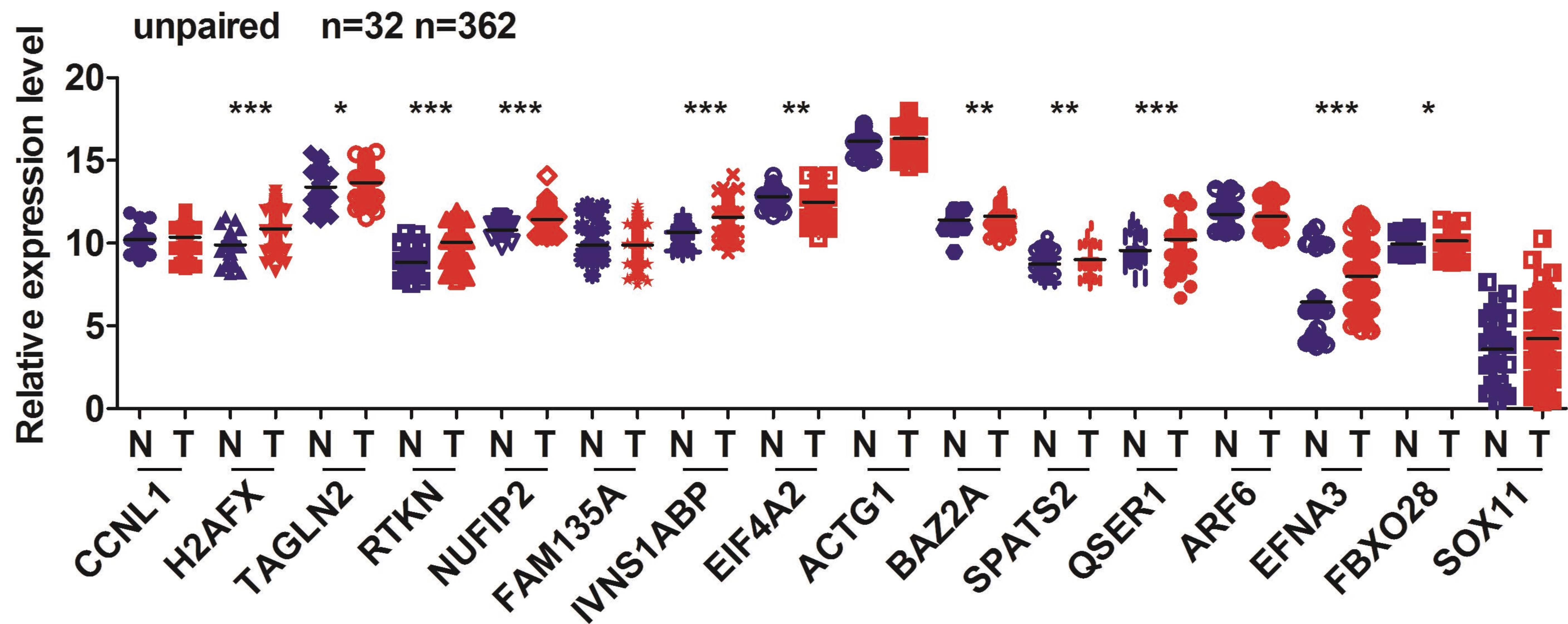

Supplement: Supplementary file 6 — Supplementary file6 (PDF 1760 kb) [file 10120_2019_1018_MOESM6_ESM.pdf]

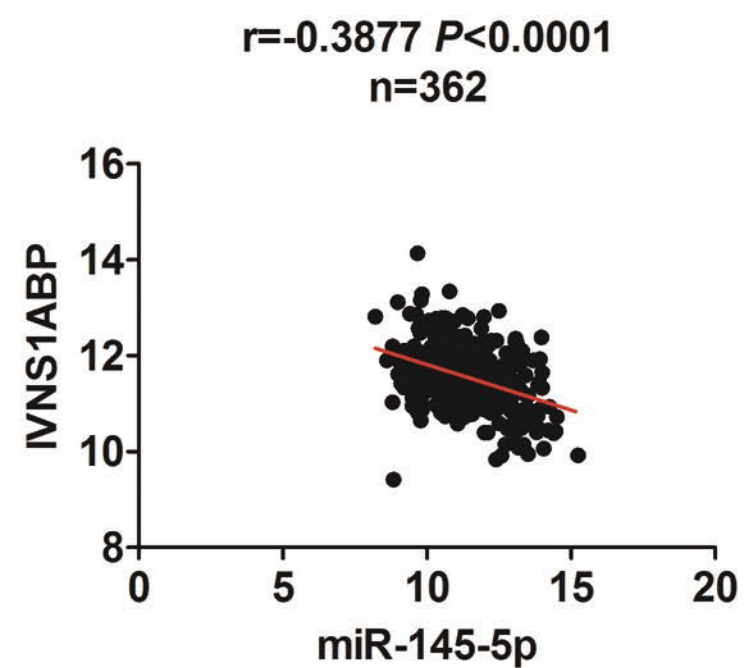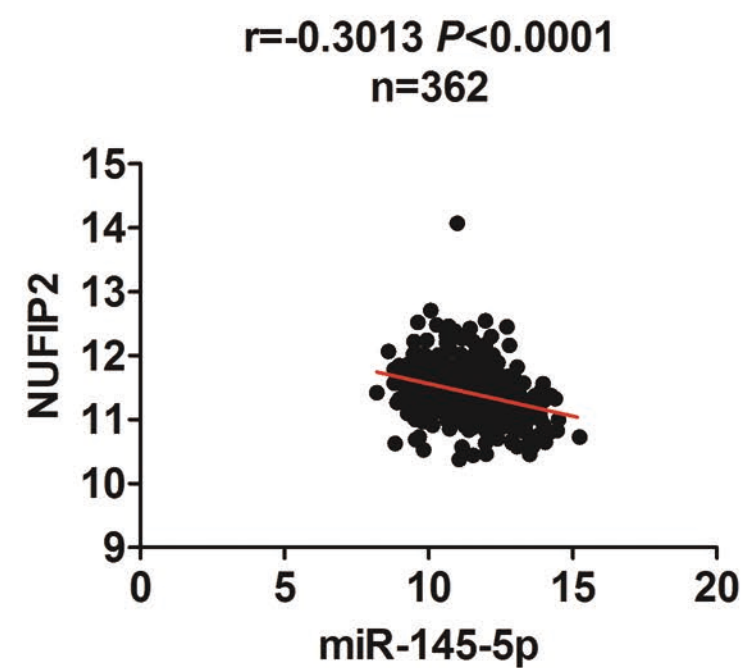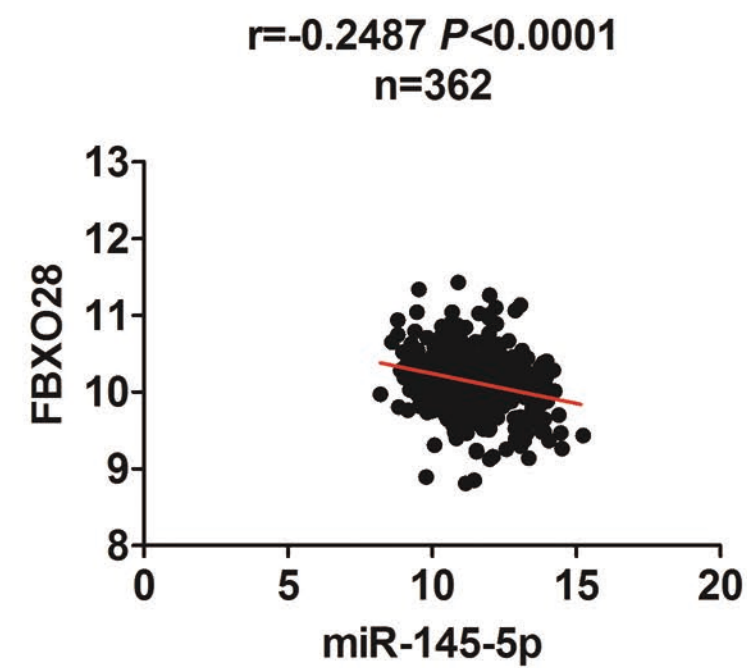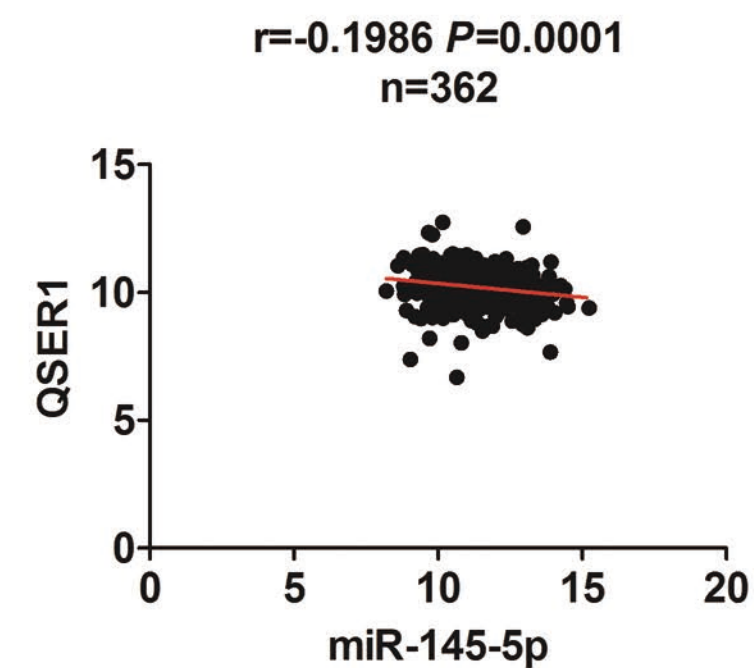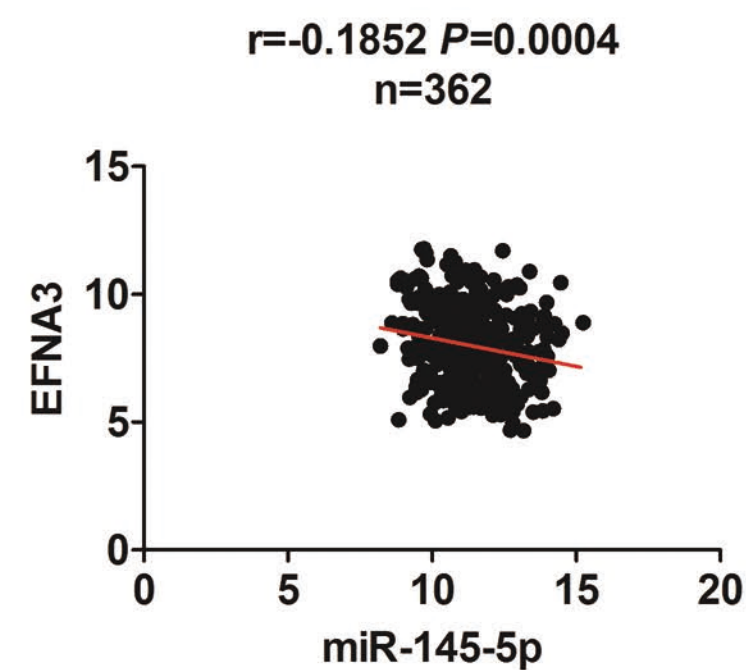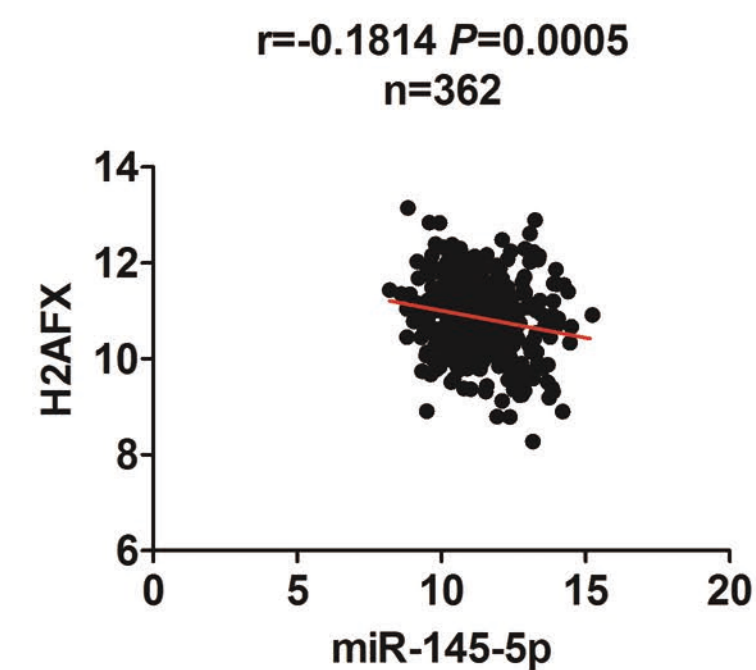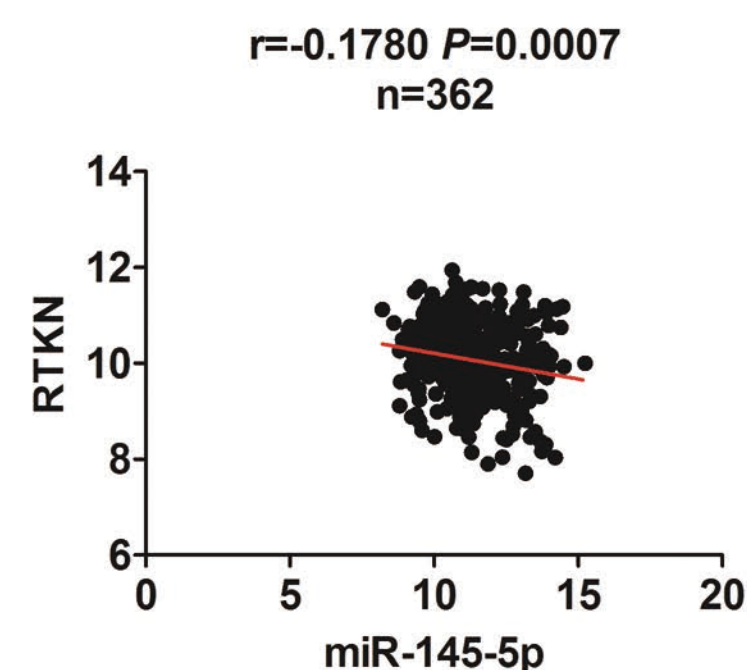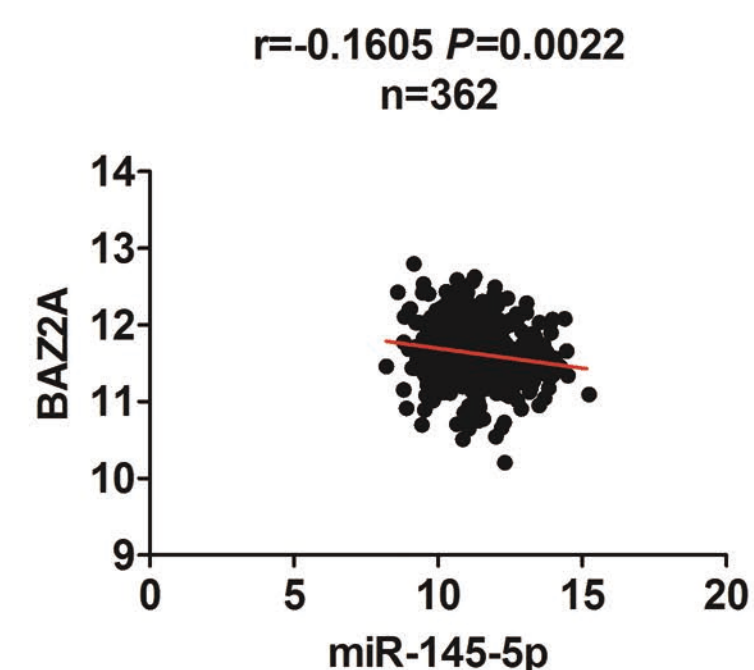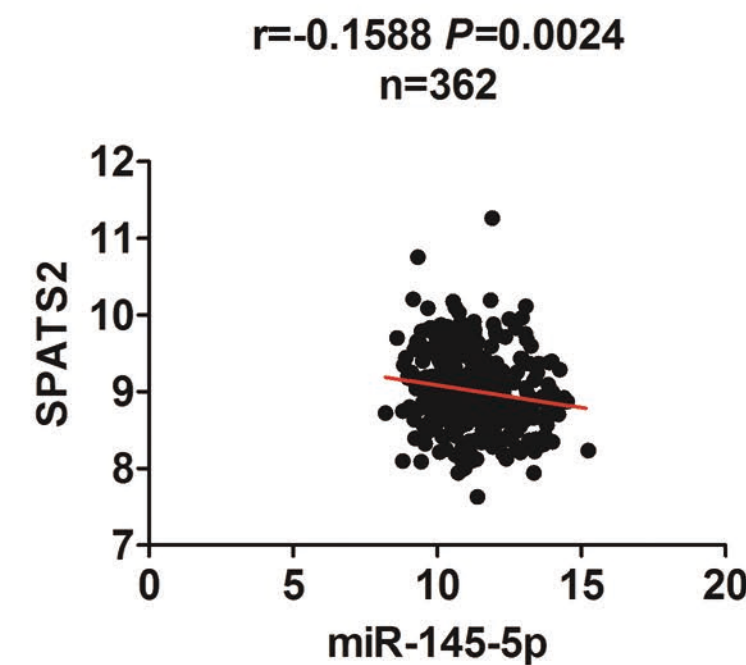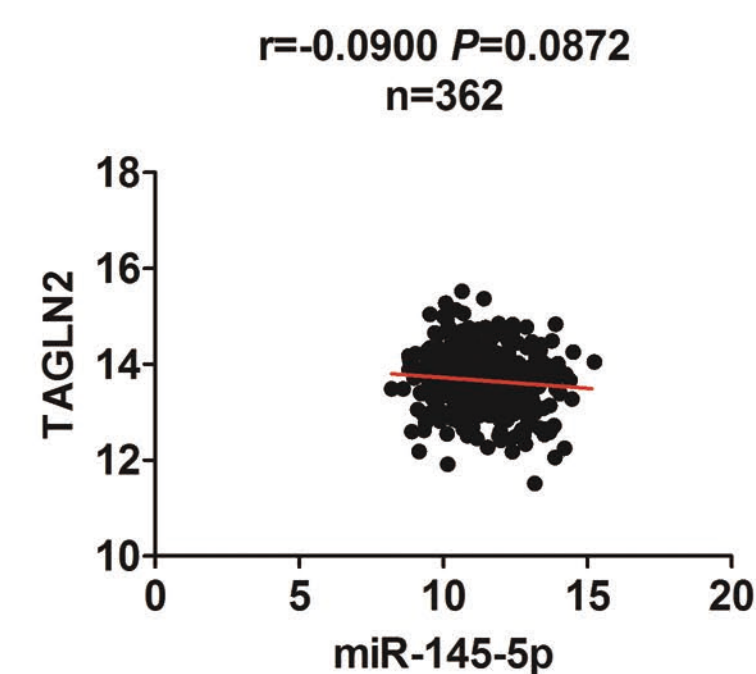

Supplement: Supplementary file 7 — Supplementary file7 (PDF 1811 kb) [file 10120_2019_1018_MOESM7_ESM.pdf]
